# Genome-wide analysis reveals the emergence of multidrug resistant *Stenotrophomonas acidaminiphila* strain SINDOREI isolated from a patient with sepsis

**DOI:** 10.3389/fmicb.2022.989259

**Published:** 2022-09-23

**Authors:** Ying Zhang, Danhua Li, Qun Yan, Ping Xu, Wei Chen, Hongya Xin, Dengshu Wu, Mingxiang Zhou, Yajing Xu, Ao Zhang, Wenjia Wei, Zhiping Jiang

**Affiliations:** ^1^Department of Hematology, Xiangya Hospital, Central South University, Changsha, China; ^2^National Clinical Research Center for Geriatric Diseases, Xiangya Hospital, Changsha, China; ^3^Hunan Hematology Oncology Clinical Medical Research Center, Changsha, China; ^4^National Clinical Research Center for Hematologic Diseases, The First Affiliated Hospital of Soochow University, Suzhou, China; ^5^Departmant of Scientific Affairs, Hugobiotech Co. Ltd., Beijing, China; ^6^Department of Laboratory Medicine, Xiangya Hospital, Central South University, Changsha, China; ^7^Department of Pharmacy, The Second Xiangya Hospital, Central South University, Changsha, China; ^8^Institute of Clinical Pharmacy, Central South University, Changsha, China; ^9^Department of Gastroenterology, Changsha Central Hospital, Changsha, China

**Keywords:** *Stenotrophomonas acidaminiphila*, sepsis, genome sequencing, genome comparisons, virulence factors, multidrug resistant, human infection

## Abstract

*Stenotrophomonas acidaminiphila*, the most recent reported species in genus *Stenotrophomonas*, is a relatively rare bacteria and is an aerobic, glucose non-fermentative, Gram-negative bacterium. However, little information of *S. acidaminiphila* is known to cause human infections. In this research, we firstly reported a multidrug-resistant strain *S. acidaminiphila* SINDOREI isolated from the blood of a patient with sepsis, who was dead of infection eventually. The whole genome of strain SINDOREI was sequenced, and genome comparisons were performed among six closely related *S. acidaminiphila* strains. The core genes (2,506 genes) and strain-specific genes were identified, respectively, to know about the strain-level diversity in six *S. acidaminiphila* stains. The presence of a unique gene (narG) and essential genes involved in biofilm formation in strain SINDOREI are important for the pathogenesis of infections. Strain SINDOREI was resistant to trimethoprim/sulfamethoxazole, ciprofloxacin, ofloxacin, cefepime, ceftazidime, and aztreonam. Several common and specific antibiotic resistance genes were identified in strain SINDOREI. The presence of two *sul* genes and exclusive determinants GES-1, *aad*A3, *qac*L, and *cml*A5 is responsible for the resistance to multidrug. The virulence factors and resistance determinants can show the relationship between the phenotype and genotype and afford potential therapeutic strategies for infections.

## Introduction

The genus *Stenotrophomonas*, which was first described with the type species *Stenotrophomonas maltophilia* (Palleroni and Bradbury, 1993), currently comprises 16 validly described species (Wang et al., 2018). Members of the genus *Stenotrophomonas* demonstrated great metabolic versatility and intraspecific heterogeneity (Ryan et al., 2009). The most recently reported species, *Stenotrophomonas acidaminiphila*, is an aerobic, glucose non-fermentative, Gram-negative bacterium, which is initially isolated from a petrochemical wastewater treated by an upflow anaerobic sludge blanket (UASB) reactor (Assih et al., 2002), and strains of *S. acidaminiphila* occur ubiquitously in the environment (Vinuesa and Ochoa-Sánchez, 2015; Patil et al., 2016; Huang et al., 2018; Huyan et al., 2020). However, the information on the characteristic of strains is still limited. To date, the reported strains of *S. acidaminiphila* were limited, especially clinical isolates. Additionally, the further genomic analysis was necessary to dig the features of *S. acidaminiphila.*

*Stenotrophomonas* species, especially *S. maltophilia* and *S. acidaminiphila*, can cause human infections (Looney et al., 2009; Huang et al., 2018). Multidrug-resistant (MDR) strains of *Stenotrophomonas* are associated with a high rate of mortality in immunocompromised patients (Paez and Costa, 2008). In the genomes of *Stenotrophomonas*, various genes encoding virulence determinants are involved in the infections (Trifonova and Strateva, 2019). Surveillance of the presence of virulence genes is important to supplement knowledge about the pathogenesis of infections (Windhorst et al., 2002; de Oliveira-Garcia et al., 2003).

Trimethoprim/sulfamethoxazole (TMP/SMX) was considered as the first-line therapy (Abbott and Peleg, 2015; Kumar et al., 2020), but was plagued by increasing resistance worldwide (Al-Jasser, 2006; Tan et al., 2008; Looney et al., 2009). Fluoroquinolones and β-lactam drugs have been used as potential alternative antibiotics to TMP/SMX for *Stenotrophomonas* infections. However, recent studies have revealed a trend in decreasing susceptibility (Wei et al., 2016; Ko et al., 2019). The strains’ intrinsic and acquired mechanisms of antibiotic resistance to most antibiotics limited the antimicrobial options for *Stenotrophomonas* infections.

The molecular mechanisms involved in its extensive antimicrobial resistance include efflux pumps and encoded genes. The *sul*1 gene, *sul*2 gene and *dfrA* gene are well known to be responsible for resistance to TMP/SMX (Barbolla et al., 2004; Domínguez et al., 2019). Two chromosomal-mediated β-lactamases, namely L1 and L2, with several regulatory genes, such as *ampR*, *ampN* and *ampG*, are associated with β-lactam resistance (Okazaki and Avison, 2008; Huang et al., 2010; Lin et al., 2011). A chromosomally encoded *qnr* gene protects both gyrase and topoisomerase IV from quinolones and confers resistance to fluoroquinolone (Ko et al., 2019). Moreover, efflux pumps are shown to be associated with resistance to multidrug and are classified to five families, namely the resistance-nodulation-cell-division (RND) family, the major facilitator superfamilies (MFS), the small multidrug resistance (SMR) family, the ATP binding cassette (ABC) family, and the multidrug and toxic compound extrusion (MATE) family (Putman et al., 2000).

To our best knowledge, *S. acidaminiphila* was mostly isolated from aquatic environments. It is worth noting that the first clinical isolate *S. acidaminiphila* SUNEO (Huang et al., 2018) was isolated from the bile of a cholangiocarcinoma patient with obstructive jaundice and cholangitis and was found resistant to sulfamethoxazole and imipenem based on the antimicrobial susceptibility testing. Meanwhile, the comparisons and analysis of whole genomes aid the identification of resistant determinants to develop the antimicrobial strategies.

In this study, the first MDR clinical isolate, *S. acidaminiphila* SINDOREI, was cultured and isolated from the blood of a patient with sepsis in China. The complete genome of SINDOREI was assembled to highlight the virulence factors and resistant genes characterizing the specific isolates. Additionally, we profiled the adaptive changes in *S. acidaminiphila* SINDORI through the characteristic of genome and genomic comparisons of six *S. acidaminiphila* strains.

## Materials and methods

### Bacterial isolation and culture conditions

Pure strain SINDOREI was cultured from the blood of a patient with sepsis. The 51-year-old man was transferred to Xiangya Hospital of Central South University (Changsha, China) on March 16, 2020, complaining of intermittent fever and full-body pain for more than 1 month without obvious causes. Based on physical examination, laboratory test results and other related examinations including bone marrow (BM) aspirate smear, flow cytometry and RT-PCR, etc., a diagnosis of sepsis was made. The antibiotic treatment did not improve his condition. The patient had a continuous high fever (39.5°C) with some new symptoms such as chills, high fever, appetite, fatigue, and shortness of breath and eventually died for multiple organ failure. The detailed medical record of the patient was shown in Supplementary Table S1. The blood samples of this patient were inoculated on nutrient agar with 5% sheep blood and incubated aerobically at 37°C overnight for three times. Only smooth, opaque and yellow colonies showing clear zones were isolated. Then, the isolates were conducted species identification by using MALDI Biotyper (Bruker, Germany) and returned no match in the database. At last, the purified isolate was further performed the whole genome sequencing and was classified as *S. acidaminiphila*.

### Antimicrobial susceptibility testing

Antimicrobial susceptibility testing was performed by VITEK2 system (bioMerieux, France) for minimum inhibitory concentration (MIC) according to the manufacturer’s instructions. MIC breakpoint was determined referring to the clinical and laboratory standards institute (CLSI) guidelines (M100, 30th Ed.; Wayne, 2020).

### Phylogenetic analysis based on the 16S rRNA sequence

The phylogenetic tree based on the 16S rRNA sequence which recovered from the genome of SINDOREI were constructed using MEGA version 11 software using maximum-likelihood method with 1,000 bootstrap replications (Tamura et al., 2021). The phylogenomic analysis based on whole genomes of the members of genus *Stenotrophomonas* were performed using the genome taxonomic database toolkit (GTDB-Tk; Chaumeil et al., 2019) and iqtree (Nguyen et al., 2015).

### Genome sequencing, assembly and annotation

The genomic DNA of *S. acidaminiphila* SINDOREI was extracted using cetyltrimethylammonium bromide (CTAB)-based methods. Then genomic DNA was randomly fragmented and was selected to average size of 200–400 bp. Adaptors were ligated to the ends of fragments by PCR assay. PCR products were processed into the sequencing library. The qualified BGI-seq libraries were sequenced on a BGISEQ-500 platform with read length of PE100. The Nanopore libraries were prepared and sequenced according to the manufacturer’s instructions (Oxford Nanopore, Oxford, UK) and sequenced on MinION with flowcell version of R9.4.1. The short reads and nanopore long reads were assembled using software Unicycler (Wick et al., 2017). The genes were predicted using Glimmer software (Delcher et al., 2007) and were functionally annotated by querying eggNOG, NR, Pfam and Swiss-Prot databases to obtain the corresponding annotations.

### SNP/InDel identification

Firstly, the genomes were broken into 500,000 reads with length of 150 bp using wgsim (v1.11)^1^ with parameters of (-e 0-r 0-R 0 -X 0). Then, these reads were mapped to SINDOREI genome using bwa (0.7.17-r1188)^2^ with default parameters. Next, the SNPs were identified by bcftools^3^ and variations with quality <20 were filtered.

### Average nucleotide identity values and digital DNA–DNA hybridization values

Average nucleotide identity (ANI) values between any two genomes were calculated using fastANI (Jain et al., 2018). The digital DNA–DNA hybridization (dDDH) values were obtained by means of genome-to-genome distance calculator *via* GGDC 3.0 using Formula 2.

### Genome comparisons and identification of core and strain-specific genes

For genome comparisons, six genomes of the *S. acidaminiphila* strains, including a type strain and other five cultured stains, were downloaded from the NCBI database (Table 1). The clusters of homologous genes among the investigated genome sequences were determined using OrthoMCL (Fischer et al., 2011). The numbers of unique core genes and strain-specific genes of all isolates were mapped. The strain-specific genes that are present in strain SINDOREI were annotated through the clusters of orthologous genes (COG) database using software EggNOG-mapper v2 (Cantalapiedra et al., 2021).

**Table 1:** The genomic information of the isolated strains of *Stenotrophomonas acidaminiphila.*

Strain	Genome size (bp)	GC content (%)	Genes (total)	CDSs (total)	Isolation source	Country	Accession number
*S. acidaminiphila* SINDOREI	3,996,619	68.73	3,623	3,536	Blood of septic patient	China	PRJCA009493
*S. acidaminiphila* SUNEO	3,660,864	69.75	3,300	3,226	Human bile	China	GCA_002951995.1
*S. acidaminiphila* ZAC14D2_NAIMI4_2	4,138,397	68.48	3,752	3,677	Sediments of polluted river	Mexico	GCA_001314305.1
*S. acidaminiphila* T25-65	3,915,662	68.96	3,556	3,481	Aerobic biofilm reactors with antibiotics	China	GCA_014076435.1
*S. acidaminiphila* T0-18	3,848,207	69.17	3,480	3,406	Aerobic biofilm reactors with antibiotics	China	GCA_014109845.1
*S. acidaminiphila* JCM13310	3,942,520	68.81	3,598	3,425	Sludge from anaerobic chemical wastewater reactor	Mexico	GCA_001431595.1

### Virulence factor and resistance genes comparisons

To analyze the virulence factors of the members of species *S. acidaminiphila*, virulence factors database (VFDB) was used (Liu et al., 2022). The protein-coding sequences were aligned against the comprehensive antibiotic resistance database (CARD; Alcock et al., 2020) and resistance-related genes were analyzed. Comparisons of specific genes related to resistance genes and efflux pumps between *S. acidaminiphila* strains were proceeded using local BLASTP alignment.

### Phylogeny based on the amino acid of *sul* genes

The phylogenetic tree based on the amino acid of *sul* genes was also constructed by MEGA version 11 software, using maximum-likelihood method with 1,000 bootstrap replications (Tamura et al., 2021). The amino acid sequences of dihydropteroate synthase from *Stenotrophomonas* strain were downloaded from the National Center for Biotechnology Information.

## Results

### Genomic features of *Stenotrophomonas acidaminiphila* SINDOREI

**Figure 1:**
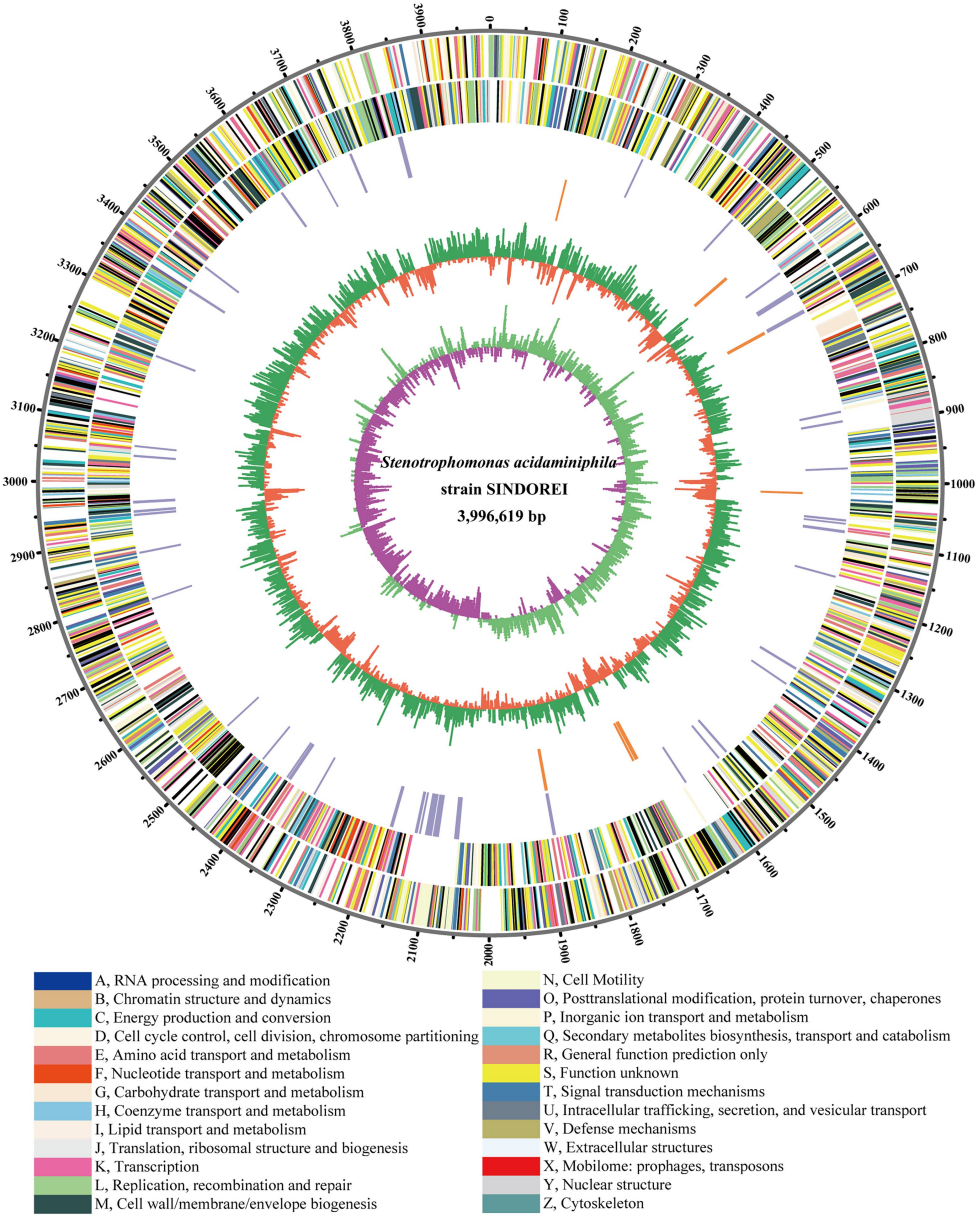
Circular plot of *Stenotrophomonas acidaminiphila* SINDORE genome generated by circos. Predicted Coding Sequences (CDSs) are presented by various colors according to cellular functions. The Circles from outside to inside: 1, the scale line. 2, CDSs on forward strands. 3, CDSs on reverse strands. 4, virulent genes. 5, resistant genes. 6, GC plot, above average in green and below average in violet, respectively. 7, GC skew showing regions above and below average in yellow and light blue, respectively.

The complete genome of SINDOREI was assembled into one circular chromosome with a total size of 3,996,619 bp and a GC content of 68.73%. SINDOREI genome contained no plasmid, and the details of the genome were listed in Supplementary Tables S2, S3. The genome of SINDOREI were functionally annotated in databases: eggNOG (Supplementary Table S4), NR (Supplementary Table S5), Pfam (Supplementary Table S6) and Swiss-Prot (Supplementary Table S7). A total of 3,623 genes, including 3,536 coding sequences (CDSs), were predicted in the genome, along with 68 transfer RNA (tRNA genes) and nine ribosomal RNA (rRNA genes). The strain SINDOREI genome was profiled as a circular map, exhibiting CDSs, virulent genes, GC plot, and GC skew (Figure 1).

### Phylogenetic analysis of members of the genus *Stenotrophomonas*

Firstly, a 16S rRNA phylogenetic tree of strain SINDOREI and other available type strains of genus *Stenotrophomonas* were constructed using the maximum-likelihood method (Supplementary Figure S1). Strain SINDORE was clustered to the branch of *S. acidaminiphila* with a bootstrap consistency of 99%. In addition, the 16S rRNA identities between SINDORE and other *S. acidaminiphila* strains was over 99.68%, indicating the strain SINDOREI belongs to *S. acidaminiphila*.

**Figure 2:**
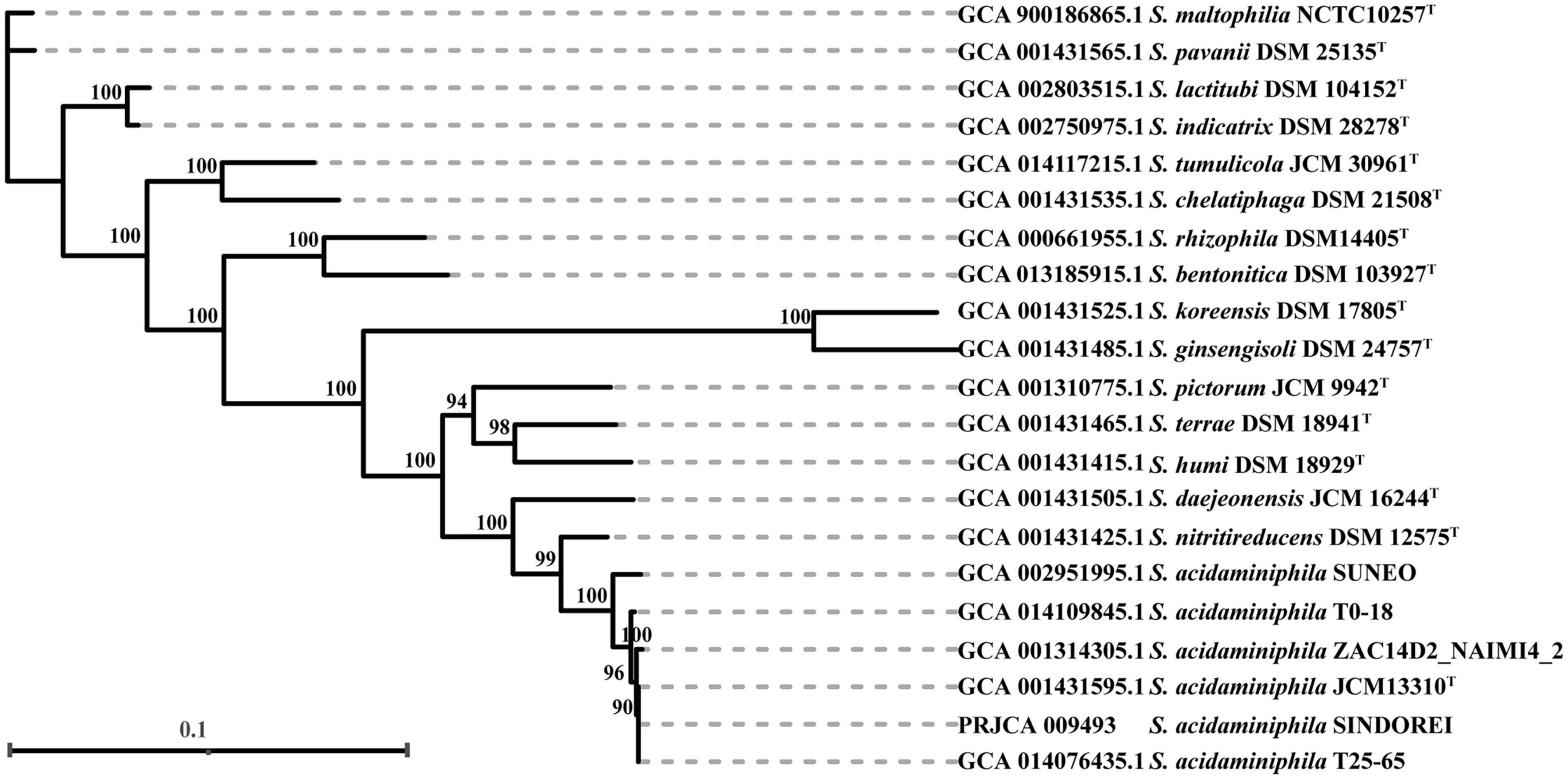
The phylogenomic tree based on the genomes of members in genus *Stenotrophomonas*. The numbers presenting on the branches of the tree represented the bootstrap values (based on 1,000 replicates). The scale bar indicated 0.1 substitutions per nucleotide position.

**Figure 3:**
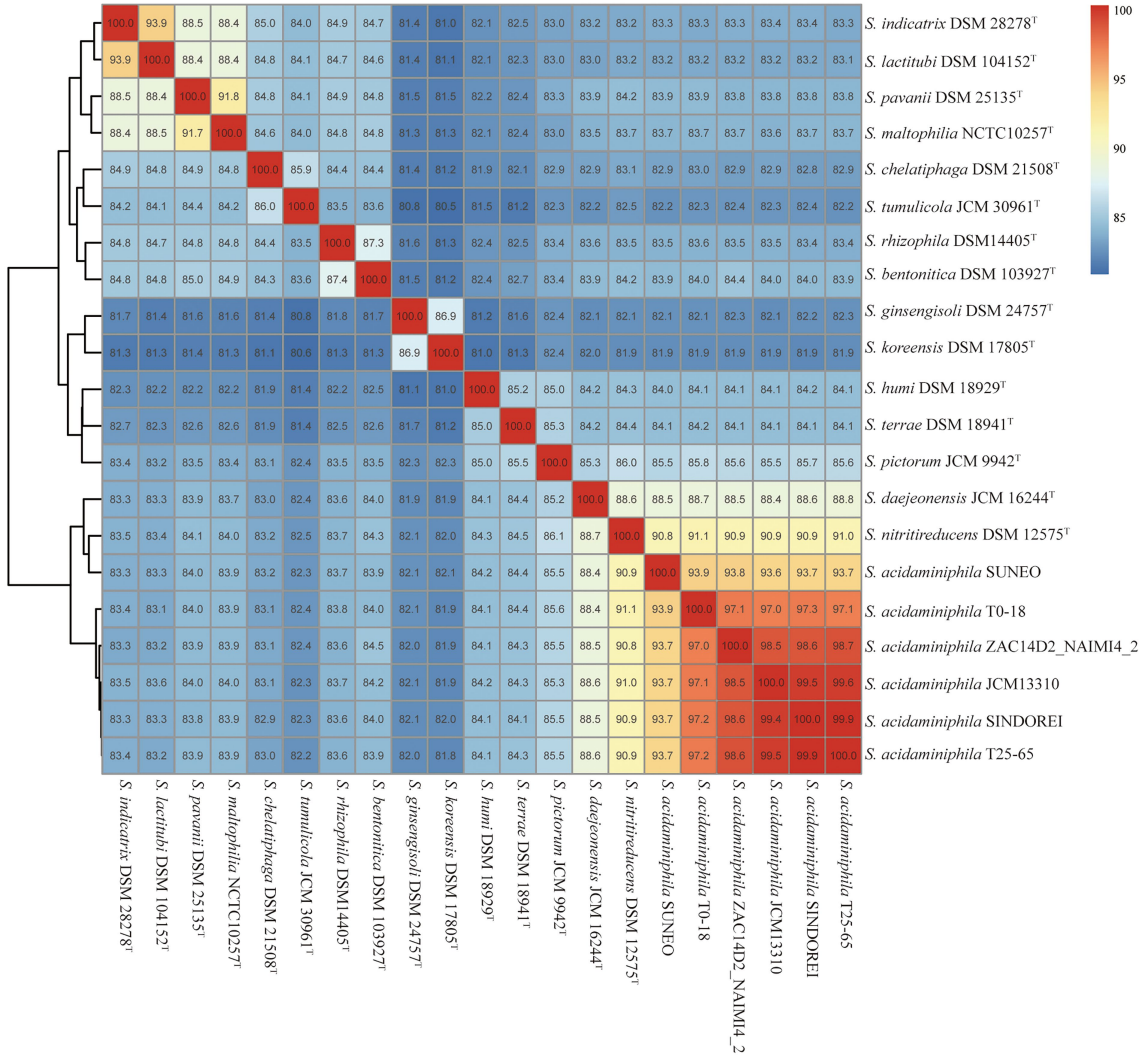
ANI values of *Stenotrophomonas acidaminiphila* SINDOREI with the type strains of genus *Stenotrophomonas*.

The phylogenomic tree was constructed and revealed that strain SINDOREI has the closest relationship with strain JCM13310^T^ and strain T25-65, which was consistent with the 16S rRNA tree (Figure 2). Whole genome comparison identified the least SNPs/Indels (11,650) between SINDOREI and T25-65 (Supplementary Table S8). Meanwhile, the ANI and dDDH values between *S. acidaminiphila* SINDOREI and *S. acidaminiphila* T25-65 are up to 99.9% and 94.1%, respectively (Figure 3; Supplementary Table S9).

### Comparative genome analysis of *Stenotrophomonas acidaminiphila*

**Figure 4:**
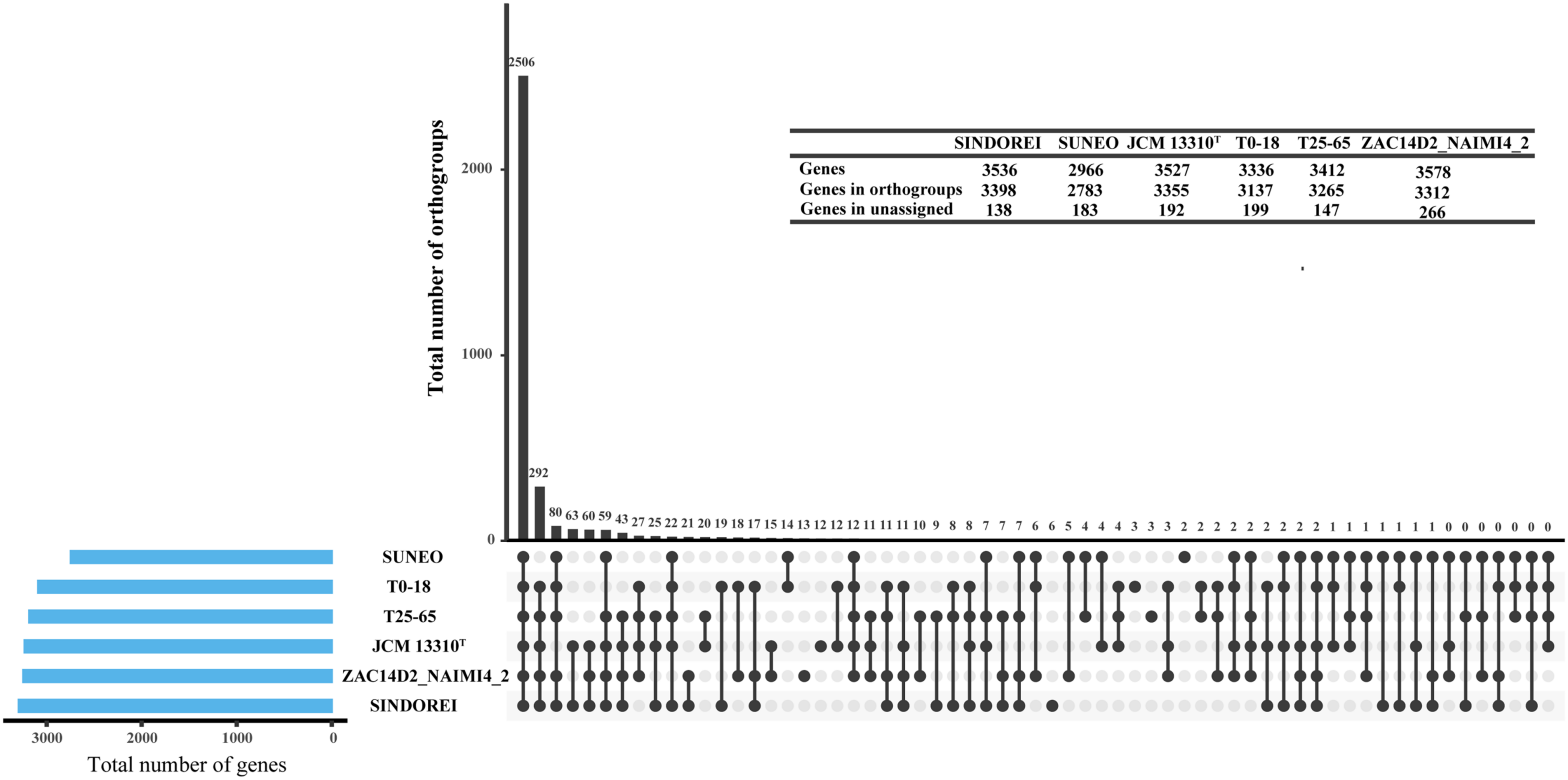
Groups of orthologous and paralogous genes (i.e., orthogroups) identified in the six *Stenotrophomonas acidaminiphila* strains used in this study. The vertical bars show the number of orthogroups exclusive to the strains marked as lower dots in the matrix. Horizontal bars represent the total number of genes in each strain.

**Figure 5:**
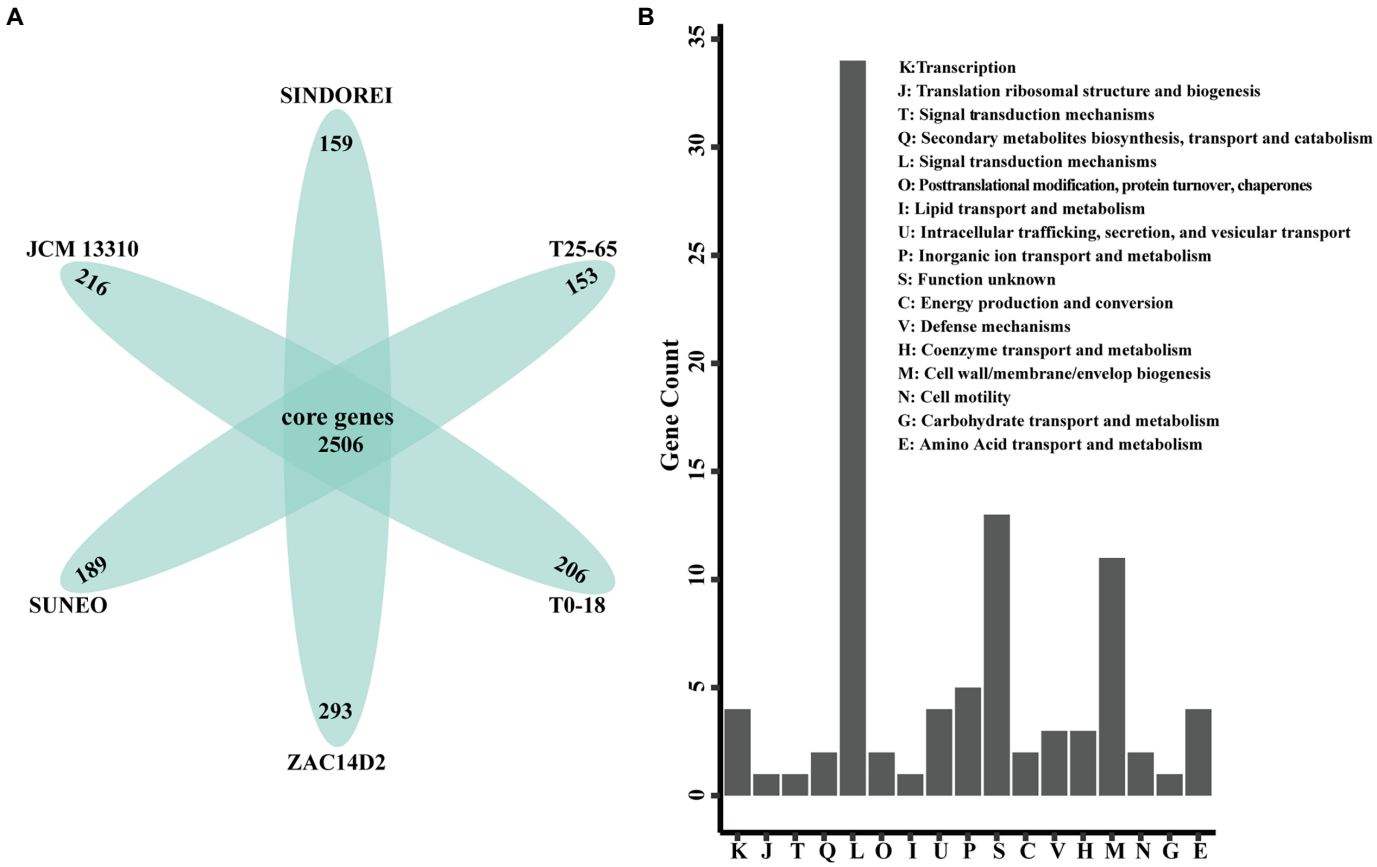
Comparison of the gene contents in *Stenotrophomonas acidaminiphila*. **(A)** Flower plot diagram showing the core genes and specific genes. **(B)** Distribution of COG functional annotations of SINDOREI specific genes.

Six genomes of *S. acidaminiphila* strains were employed for the comparative genome analysis (Table 1). Only SINDOREI and SUNEO were isolated from clinical specimens. The largest genome size was from ZAC14D2_NAIMI4_2 (4,138,397 bp), followed by strain SINDOREI. There were 2,506 orthogroups found in each strain (Figure 4), which represented the set of non-redundant core genes of six strains (Figure 5). The second highest orthogroups (292 orthogroups) were uniquely observed in SINDOREI, JCM13310^T^, T25-65, T0-18 and ZAC14D2_NAIMI4_2, except SUNEO. Further analysis of pair-wise comparisons demonstrated that strain SINDOREI share 3,166, 3,105, 3,084, 3,028, and 2,691 orthogroups with T0-18, JCM 13310^T^, T25-65, ZAC14D2_NAIMI4_2 and SUNEO, respectively (Supplementary Figure S2). Strain ZAC14D2_NAIMI4_2 had the most strain-specific genes (293 genes) as shown in Figure 5A, followed by strain JCM 13310^T^ (216 genes). The ratio of specific genes in strain ZAC14D2_NAIMI4_2, SUNEO, T0-18, JAM 13310^T^, SINDOREI, and T25-65 was 8.2%, 6.4%, 6.2%, 6.1%, 4.5%, and 4.5%, respectively. 159 genes were exclusive to strain SINDOREI, and functional analysis based on COG category revealed that these genes were mainly assigned to signal transduction mechanisms (Figure 5B). The annotations of SINDOREI specific orthogroups and genes were listed in Supplementary Table S10.

### Virulence factors associated with infections

**Figure 6:**
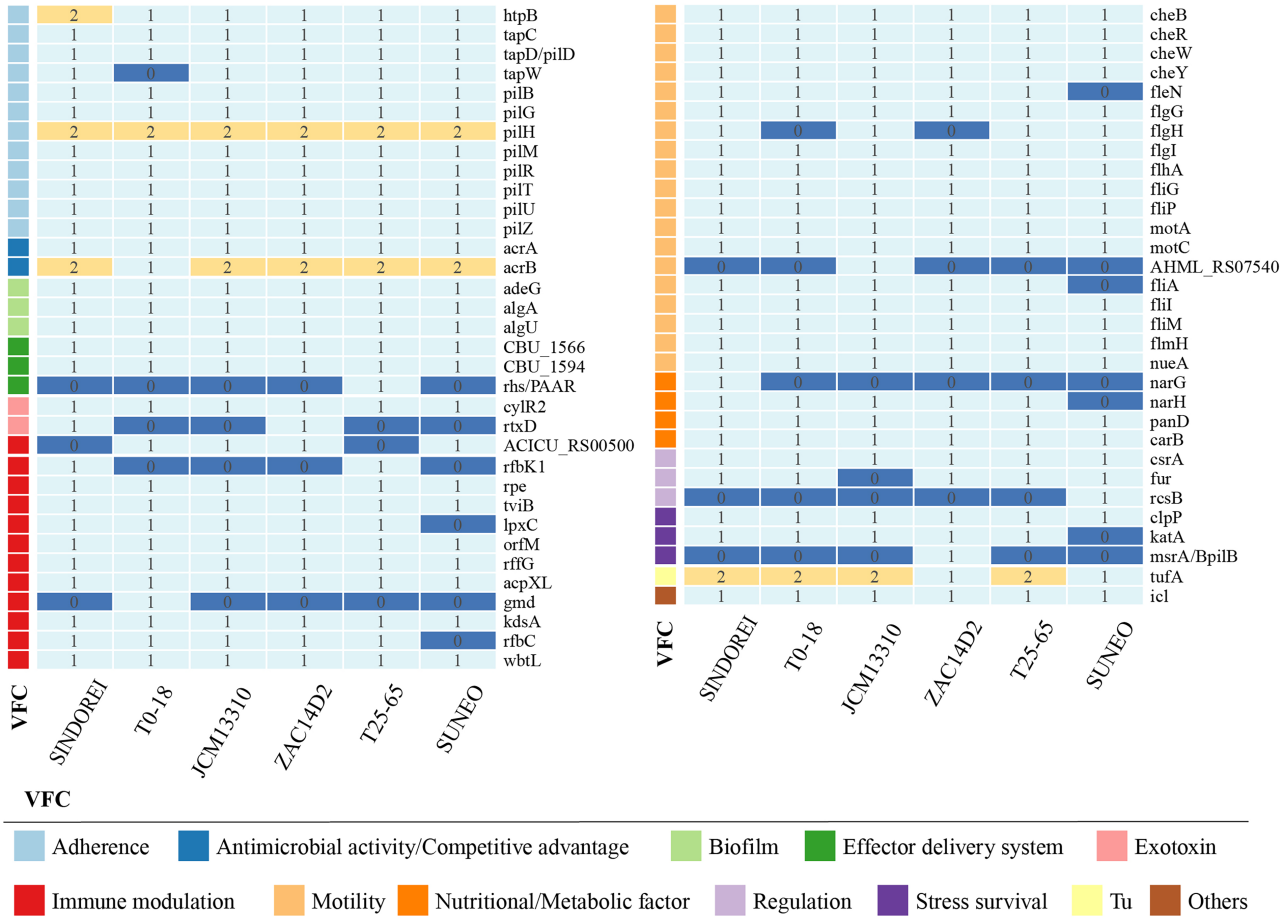
Comparison of the virulence factor genes in *Stenotrophomonas acidaminiphila.* The numbers show the gene copies in each genome and the virulence factor genes were classified into categories.

Virulence factors are components, produced by bacterial cells, which generally cause damages to the host by increasing adhesion, facilitating colonization and invasion into eukaryotic cells, escaping the host immune responses and providing the essential nutrient (Casadevall and Pirofski, 2009). The presence of various virulence genes was investigated in the strain SINDOREI genome and the comparison of virulence genes between related strain genomes was conducted. The results revealed that the virulence genes in *S. acidaminiphila* genomes are classified into 11 categories according to virulence factor (VF) category, including motility (flagella), adherence (Type IV pili and non-pilus adhesins), biofilm formation, immune modulation [lipooligosaccharides (LOS), capsule, and lipopolysaccharide (LPS)], antimicrobial activity/competitive advantage, stress survival, nutritional/metabolic factor, regulation, exotoxin, effector delivery system, and others. The virulence genes of *S. acidaminiphila* genomes was shown in Figure 6 and the details of were listed in Supplementary Table S11. The genomes of *S. acidaminiphila* strains included the same VF categories, but the presence and distribution of genes were different. It is worth noting that the *narGH* operon only found in strain SINDOREI is responsible for nitrate reductase, which is identified as an important virulence factor for many bacterial infections, such as *Mycobacterium tuberculosis* and *Pseudomonas aeruginosa* (Palmer et al., 2007; Sohaskey and Modesti, 2009).

Biofilm formation, which is a mixture of cells, polysaccharides, nucleic acids, lipids and proteins, provides resistance to various antimicrobial drugs and to host immune defense of bacterial virulence. Biofilm-related infections represent more than 60% of all microbial infections in humans. In the genome of strain SINDOREI, various virulence factors are involved in the biofilm formation according to the previously reported including LPS (Huang et al., 2006; Pompilio et al., 2011; Zhuo et al., 2014; Madi et al., 2016), flagella formation (Di Bonaventura et al., 2007; Kang et al., 2015), type IV pili (Strom and Lory, 1993; Giltner et al., 2012), and purine biosynthesis (Kang et al., 2015). Details are listed in Table 2. These genes associated with biofilm formation exist in the genome of strain SINDOREI, suggesting strain SINDOREI harbor the basic characteristic of biofilm formation.

**Table 2:** Genes involved in biofilm formation in *Stenotrophomonas acidaminiphila* SINDOREI.

Genes	SINDOREI gene locus	Activity	Function	References
Polysaccharides				
*wbtL*	JNIIIPNH_00556	Lipopolysaccharides Biosynthesis invovled in Biofilm formation	Glucose-1-phosphate thymidyl transferase	Huang et al. (2006), Pompilio et al. (2011), Zhuo et al. (2014), and Madi et al. (2016)
*rfbC*	JNIIIPNH_00557		dTDP-4-dehydrorhamnose 3,5-epimerase	
*spgM*	JNIIIPNH_00560		Phosphoglucomutase/phosphomannomutase	
Flagella				
*flgG*	JNIIIPNH_01883	Flagella formation. Flagella-mediated attachment	Flagellar basal body rod protein	Di Bonaventura et al. (2007) and Kang et al. (2015)
*flgH*	JNIIIPNH_01884		Flagellar basal body L-ring protein precursor	
*flgI*	JNIIIPNH_01885		Flagellar basal body P-ring protein precursor	
*flhA*	JNIIIPNH_01923		Flagellar biosynthesis protein	
*fliI*	JNIIIPNH_01911		Flagellum-specific ATP synthase	
*fliM*	JNIIIPNH_01915		Flagellar motor switch protein	
*fliN*	JNIIIPNH_01916		Flagellar motor switch protein	
*fliA*	JNIIIPNH_01926		Flagellar biosynthesis sigma factor	
Fimbriae				
*pilU*	JNIIIPNH_00520	Type IV pili formation. Type IV pili and twitching motility associated with biofilm formation	Twitching motility	Strom and Lory (1993) and Giltner et al. (2012)
*pilU*	JNIIIPNH_00971			
*pilZ*	JNIIIPNH_00959		Type 4 fimbrial biogenesis	
*pilT*	JNIIIPNH_00970		Twitching motility	
*pilH*	JNIIIPNH_02554		Twitching motility	
*pilH*	JNIIIPNH_02624			
*pilG*	JNIIIPNH_02625		Twitching motility	
*pilR*	JNIIIPNH_02689		Two-component response regulator	
*pilB*	JNIIIPNH_02692		Type 4 fimbrial biogenesis	
*tapC*	JNIIIPNH_02705		Type IV fimbrial assembly	
*tapD/pilD*	JNIIIPNH_02706		Prepilin peptidase	
*pilM*	JNIIIPNH_02777		Type IV pilus inner membrane	
*tapU*	JNIIIPNH_00971		Twitching ATPase	
Other				
*purD*	JNIIIPNH_03072	Purine biosynthesis involved in biofilm formation	Phosphoribosylamine-glycine ligase	Kang et al. (2015)
*purC*	JNIIIPNH_03118		Phosphoribosylaminoimidazolesuccinocarboxamide	
*purI*	JNIIIPNH_02993		Phosphoribosylformylglycinamidine synthase	

### Comparative analysis of antibiotic resistance genes

Antimicrobial susceptibility test revealed that strain SINDOREI is resistant to six antibiotics including TMP/SMX, ciprofloxacin, ofloxacin, cefepime, ceftazidime, and aztreonam (Table 3), which are classified as sulfonamide antibiotic, fluoroquinolone antibiotic and β-lactam antibiotic. At the same time, strain SINDOREI is sensitive to meropenem, piperacillin tazobactam, sulbactam cefoperazone, amikacin, and tigecycline.

**Table 3:** Antimicrobial susceptibility test of *Stenotrophomonas acidaminiphila* SINDOREI, SUNEO and JCM 13310^T^.

Class	Antibiotics	SINDOREI	SUNEO	JCM 13310^T^
Sulfonamide antibiotic	Trimethoprim/Sulfamethoxazole	≥64/304, R	80 (4/76), R	≤2/38, S
Fluoroquinolone antibiotic	Ciprofloxacin	≥4, R	≤0.25, S	≤1, S
	Ofloxacin	≥8, R		≤1, S
β-lactam antibiotic	Cefalotin			> 32, R
	Cefepime	≥32, R	≤1, S	
	Ceftriaxone		16, I	
	Ceftazidime	≥64, R	≤1, S	≤4, S
	Amoxicillin			> 16, R
	Piperacillin			≤16, S
	Aztreonam	≥64, R		
	Imipenem		≥16, R	> 8, R
	Meropenem	≤0.25, S		
β-lactam combination agents	Amoxicillin clavulanic acid			> 16, R
	Ampicillin Sulbactam		≤2, S	
	Piperacillin tazobactam	8, S	≤4, S	≤16, S
	Sulbactam cefoperazone	≤8, S		
Aminoglycoside antibiotic	Amikacin	16, S	16, S	≤8, S
	Gentamicin		2, S	≤4, S
Tetracycline derivative	Tigecycline	≤0.5, S	≤0.5, S	

The comparison of resistance-related genes in strains SINDOREI, SUNEO, JCM 13310^T^, T25-65, T0-18 and ZAC14D2_NAIMI4 was performed (Table 4). The results showed that *S. acidaminiphila* possesses similar antibiotic resistance genes. A total of 24 key genes including encoded genes (14 genes) and efflux pumps (10 genes) contribute to the multidrug resistance in strain SINDOREI. The 24 genes are involved in resistance to TMP/SMX (Toleman et al., 2007; Hu et al., 2011), fluoroquinolone (Valdezate et al., 2005; Sanchez et al., 2009; Farhat et al., 2016), β-lactam (Okazaki and Avison, 2008; Huang et al., 2010; Lin et al., 2011; Bontron et al., 2015), aminoglycoside (Huang et al., 2015), disinfecting agents (Huang et al., 2015), phenicol (Dong et al., 2020) and tetracycline (Kadlec and Schwarz, 2018). Interestingly, four antibiotic resistant determinants, including GES-1, *aad*A3, *qac*L and *cml*A5, were exclusive to strain SINDOREI. The *sul*2 and tetC genes were only found in SINDOREI, T25-65, and T0-18.

**Table 4:** The antibiotic resistance genes among the *Stenotrophomonas acidaminiphila* strains.

Genes	SINDOREI	SUNEO	ZAC14D2_NAIMI4_2	T25-65	T0-18	JCM13310
Trimethoprim/sulfamethoxazole resistance gene						
*sul1*	JNIIIPNH_01405	B1L07_06465	AOT14_RS07185	F0P98_RS10870	F0P95_RS03720	ABB33_13125
*sul2*	JNIIIPNH_01524	–	–	F0P98_RS10870	F0P95_RS03720	–
*dfrA*	JNIIIPNH_02899	–	AOT14_RS14395	F0P98_RS03500	F0P95_RS17150	–
Fluoroquinolone resistance gene						
*qnr*	JNIIIPNH_03313	B1L07_15000	AOT14_RS17045	F0P98_RS16470	F0P95_RS02400	–
β-lactam resistance gene						
*L1*	JNIIIPNH_02567	B1L07_11340	AOT14_RS12805	F0P98_RS05130	F0P95_RS15250	ABB33_10340
*L2*	JNIIIPNH_01026	B1L07_04670	AOT14_RS05350	F0P98_RS12890	F0P95_RS08575	ABB33_03240
*GES-1*	JNIIIPNH_00474	–	–	–	–	–
*ampR*	JNIIIPNH_01025	B1L07_04665	AOT14_RS05345	F0P98_RS12895	F0P95_RS08570	ABB33_02800
*ampN*	JNIIIPNH_00207	B1L07_01060	AOT14_RS02770	F0P98_RS01145	F0P95_RS04785	ABB33_12865
*ampG*	JNIIIPNH_00208	B1L07_01065	AOT14_RS02775	F0P98_RS01150	F0P95_RS04790	ABB33_12870
*ampD*	JNIIIPNH_02390	B1L07_01310	AOT14_RS11925	F0P98_RS06020	F0P95_RS14375	ABB33_06015
*mrcA*	JNIIIPNH_02778	B1L07_01315	AOT14_RS13800	F0P98_RS04120	F0P95_RS16525	ABB33_04710
*mrdA*	JNIIIPNH_00607	B1L07_02815	AOT14_RS03340	F0P98_RS14735	F0P95_RS06700	–
Aminoglycoside resistance gene						
*aadA3*	JNIIIPNH_01525	–	–	–	–	–
Efflux pump						
smeDEF RND system						
*sme D*	JNIIIPNH_01709	B1L07_07555	AOT14_RS08230	F0P98_RS14800	F0P95_RS06630	ABB33_10360
*sme E*	JNIIIPNH_01710	B1L07_07560	AOT14_RS08235	F0P98_RS14795	F0P95_RS06635	ABB33_10365
*sme F*	JNIIIPNH_01712	B1L07_07570	AOT14_RS08245	F0P98_RS05900	F0P95_RS14485	ABB33_10375
smeOP-TolC RND system						
*tolC*	JNIIIPNH_00706	B1L07_03300	AOT14_RS03825	F0P98_RS14240	F0P95_RS07185	ABB33_09990
*pcm*	JNIIIPNH_00707	B1L07_03305	AOT14_RS03830	F0P98_RS14235	F0P95_RS07190	ABB33_09985
*smeO*	JNIIIPNH_00709	B1L07_03315	AOT14_RS03840	F0P98_RS04655	F0P95_RS16015	ABB33_09950
*smeP*	JNIIIPNH_00710	B1L07_03320	AOT14_RS03845	F0P98_RS14220	F0P95_RS07205	ABB33_09945
SMR efflux pump						
*qacL*	JNIIIPNH_01527	–	–	–	–	–
MFS efflux pump						
*cmlA5*	JNIIIPNH_01526	–	–	–	–	–
*tetC*	JNIIIPNH_01532	–	–	F0P98_RS10910	F0P95_RS03755	–

### Homologues of the *sul* genes in *Stenotrophomonas acidaminiphila* genomes

**Figure 7:**
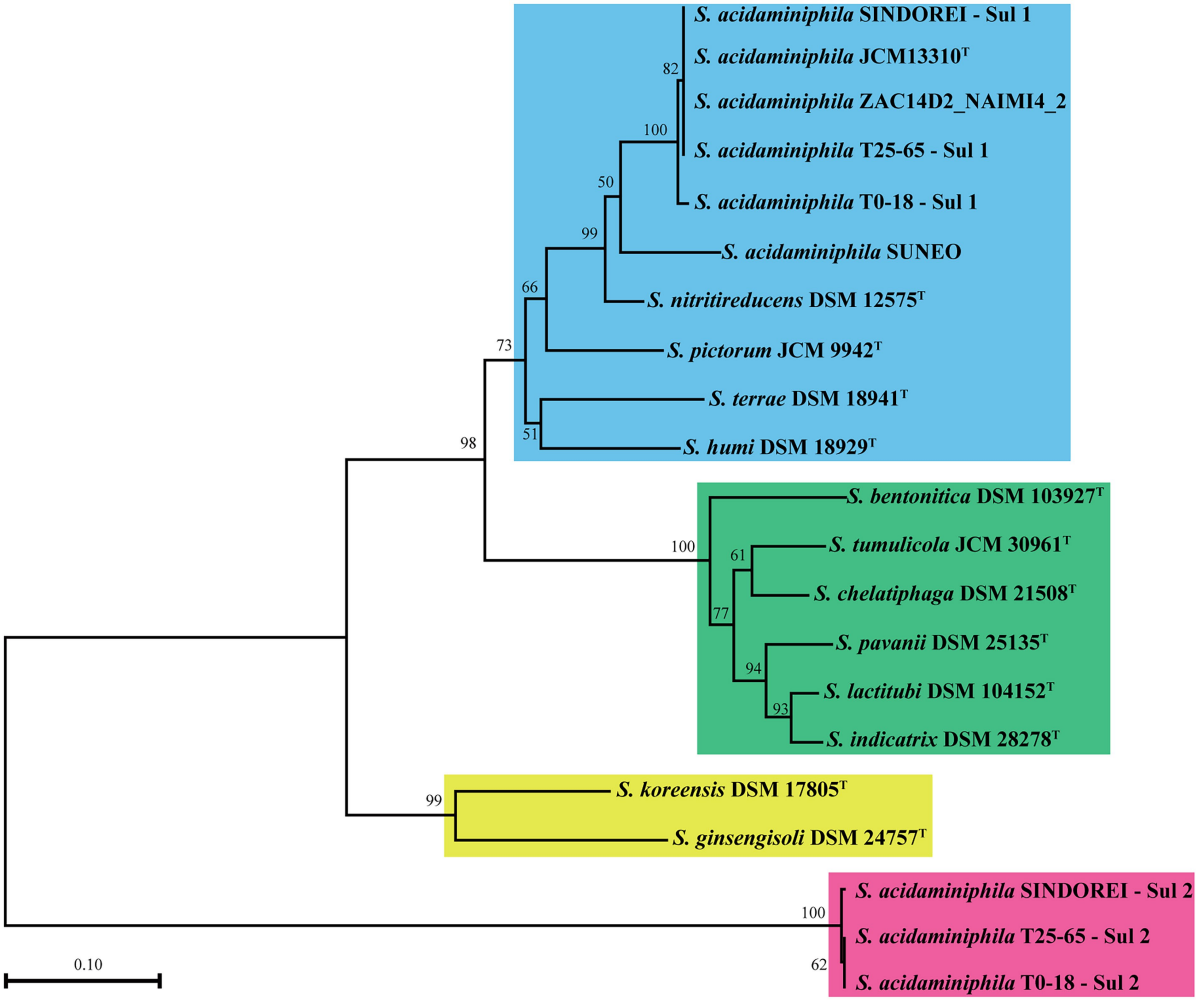
The phylogenetic tree based on the amino acid of *sul* genes in the members of genus *Stenotrophomonas*. The numbers present on the branches of the tree represent the bootstrap values (based on 1,000 replicates).

The *sul* genes encoding variants of the dihydropteroate synthase are responsible for resistance to TMP/SMX in many bacteria (Huang et al., 2018; Domínguez et al., 2019). There are two *sul* genes (*sul*1 and *sul*2 genes) in Strain SINDOREI. The amino acid sequences of homologues to the *sul* genes in *Stenotrophomonas* genomes were employed to construct the phylogenetic tree. Four distinct groups were generated (Figure 7), and Sul1 and Sul2 in SINDOREI belong to different groups. The distribution of *sul*1 and *sul*2 genes in strain SINDOREI, T0-18 and T25-65 may promote the resistance to TMP/SMX. The amino acid sequence of Sul1 in strain SINDOREI shows 100% identity with that of strains JCM13310^T^, ZAC14D2_NAIMI4_2 and T25-65. The identity sequences suggests that these elements are ancient and acquired long with the use of antibiotics. According to the genome sequence of strain SINDOREI, we noted that IS6 and Tn3 family transposase were located immediately upstream of *sul*2 and IS6 family transposase was located downstream of *sul*2, suggesting that *sul*2 gene was acquired from horizontal gene transfer.

## Discussion

In this study, the genomes of six *S. acidaminiphila* strains, including SINDOREI, SUNEO, JCM13310^T^, T25-65, T0-18, and ZAC14D2_NAIMI4_2, were employed to perform the comparative analysis. Strain SINDOREI was isolated from the blood of a patient with sepsis. Meanwhile, *S. acidaminiphila* was the sole organism cultured from this patient. Antimicrobial susceptibility test reveals that stain SINDOREI is resistant to TMP/SMX, ciprofloxacin, ofloxacin, cefepime, ceftazidime, and aztreonam. The results suggested that *S. acidaminiphila* is an emerging opportunistic pathogen with environmental origin.

Based on the analysis of six genomes, strain SINDOREI shows highest ANI and dDDH values with T25-65 and shared lowest ANI and dDDH values with SUNEO. Two thousand five hundred six orthogroups were found in six genomes, though only 2,691 orthogroups was shared between strain SINDOREI and SUNEO. The clinical isolates, strain SINDOREI and SUNEO, shows relatively high strain-level diversity compared to other strains. Further work is still needed for getting more isolates to profile a comprehensive picture of the population of *S. acidaminiphila*.

We explored the virulence features in strain SINDOREI, and the nitrate reductase enzyme operon (*narGH*) was also found. However, *narH* gene was only found in strain JCM 13310^T^, T25-65, T0-18 and ZAC14D2_NAIMI4_2. As reported previously, the nitrate reductase enzyme is important in the pathogenesis of many bacteria and a *narG* knockout mutant can cause reduced virulence and reduce lung damage in severe combined immunodeficiency mice (Fritz et al., 2002). Biofilm formation is also an important pathogenesis of bacteria such as *S. maltophilia*. Various genes associated with biofilm formation were found in strain SINDOREI which were considered as the basic characteristics of biofilm formation involved in pathogenesis of infections. Biofilm can be attached to various abiotic surfaces and tissues (Flores-Treviño et al., 2019). This specific structure provides up to 1,000 times more resistance to antimicrobial drugs (Mah, 2012; Olsen, 2015) and contributes to respiratory diseases (Costerton et al., 1999; Pompilio et al., 2010). New antimicrobial strategies (antibiofilm strategies) were used to treat *Stenotrophomonas* infections. Biofilm assay should be conducted in further study to explore biofilm’s correlations with virulence. Therefore, present ongoing studies about strain SINDOREI are limited and valuable to in-depth mining.

Investigation of the presence of virulence genes is important to explain the genetic mechanisms of multidrug resistance. In MDR strain SINDOREI, 24 genes involved in the resistance to a broad array of antimicrobial agents were analyzed. Strain SINDOREI has all the target genes mainly encoding antibiotic inactivating enzymes and multidrug efflux pumps, which is an important reason for the resistance to antibiotics. Meanwhile, the unique and redundant resistance genes occurred in strain SINDOREI. GES-1 (GES-type beta-lactamase), *aad*A3 (streptomycin 3′-adenylyltransferase), *qac*L (antibiotic efflux of disinfecting agents), and *cml*A5 (antibiotic efflux of phenicol antibiotic) associated with the resistance to β-lactam, aminoglycoside, disinfecting agents and phenicol are exclusive to strain SINDOREI. The phylogenetic analysis of homologues to *sul* genes indicated that there are two types of *sul* genes (*sul*1 and *sul*2) in strain SINDOREI. The presence of redundant genes (*sul*1 and *sul*2) in strain SINDOREI, should be responsible for the increased resistance to TMP/SMX. The presence of IS6 and Tn3 family transposase located upstream and downstream of the *sul*2, suggesting that acquisition of resistance genes is a relevant mechanism for strain SINDOREI antibiotic resistance.

## Conclusion

In this study, we reported the world’s first case of fatal infection with *S. acidaminiphila* and obtained the high-quality genome of clinical isolated MDR strain SINDOREI. The comparative genomes analysis suggested a unique gene (narG) and key genes involved in biofilm formation in strain SINDOREI played an important role in pathogenesis of infections. Antimicrobial susceptibility test revealed that stain SINDOREI were resistant to TMP/SMX, ciprofloxacin, ofloxacin, cefepime, ceftazidime, and aztreonam. The presence of redundant Sul1 and Sul2 from two distinct groups and the exclusive determinants GES-1, *aad*A3, *qac*L and *cml*A5 exist in SINDOREI, which can explain the mechanisms of strain’s multidrug resistance and afford potential therapeutic strategies for pathogen infections.

## Data availability statement

The genome sequence data of strain SINDOREI has been deposited in National Genomics Data Center (NGDC, https://ngdc.cncb.ac.cn/) and accession numbers is PRJCA009493, as mentioned in Table 1 (CNCB-NGDC Members and Partners., 2022).

## Ethics statement

Written informed consent was obtained from the individual(s) for the publication of any potentially identifiable images or data included in this article.

## Author contributions

YZ, PX, and WC made contributions to the acquisition of clinical data. QY, MZ, AZ, and WW contributed to laboratory work. DW, HX, and YX conducted the clinical work. YZ and DL drafted the manuscript. ZJ revised the manuscript critically for important intellectual content and had given final approval of the version to be published. All authors contributed to the article and approved the submitted version.

## Funding

This paper was supported by the Key Research and Development program of the Hunan Provincial Science and Technology Department (grant no. 2022SK2005).

## Conflict of interest

DL was employed by Hugobiotech Co. Ltd.

The remaining authors declare that the research was conducted in the absence of any commercial or financial relationships that could be construed as a potential conflict of interest.

## Publisher’s note

All claims expressed in this article are solely those of the authors and do not necessarily represent those of their affiliated organizations, or those of the publisher, the editors and the reviewers. Any product that may be evaluated in this article, or claim that may be made by its manufacturer, is not guaranteed or endorsed by the publisher.

## Footnotes

^1^ https://github.com/lh3/wgsim

^2^ https://github.com/lh3/bwa

^3^ https://github.com/samtools/bcftools
