# Supplementary material for: Genome-wide analysis reveals the emergence of multidrug resistant Stenotrophomonas acidaminiphila strain SINDOREI isolated from a patient with sepsis
Source: Front Microbiol. 2022 Sep 23;13:989259. doi: 10.3389/fmicb.2022.989259 (PMC9537462; doi:10.3389/fmicb.2022.989259)
Supplement: Supplementary file 1 [file Table_1.DOCX]

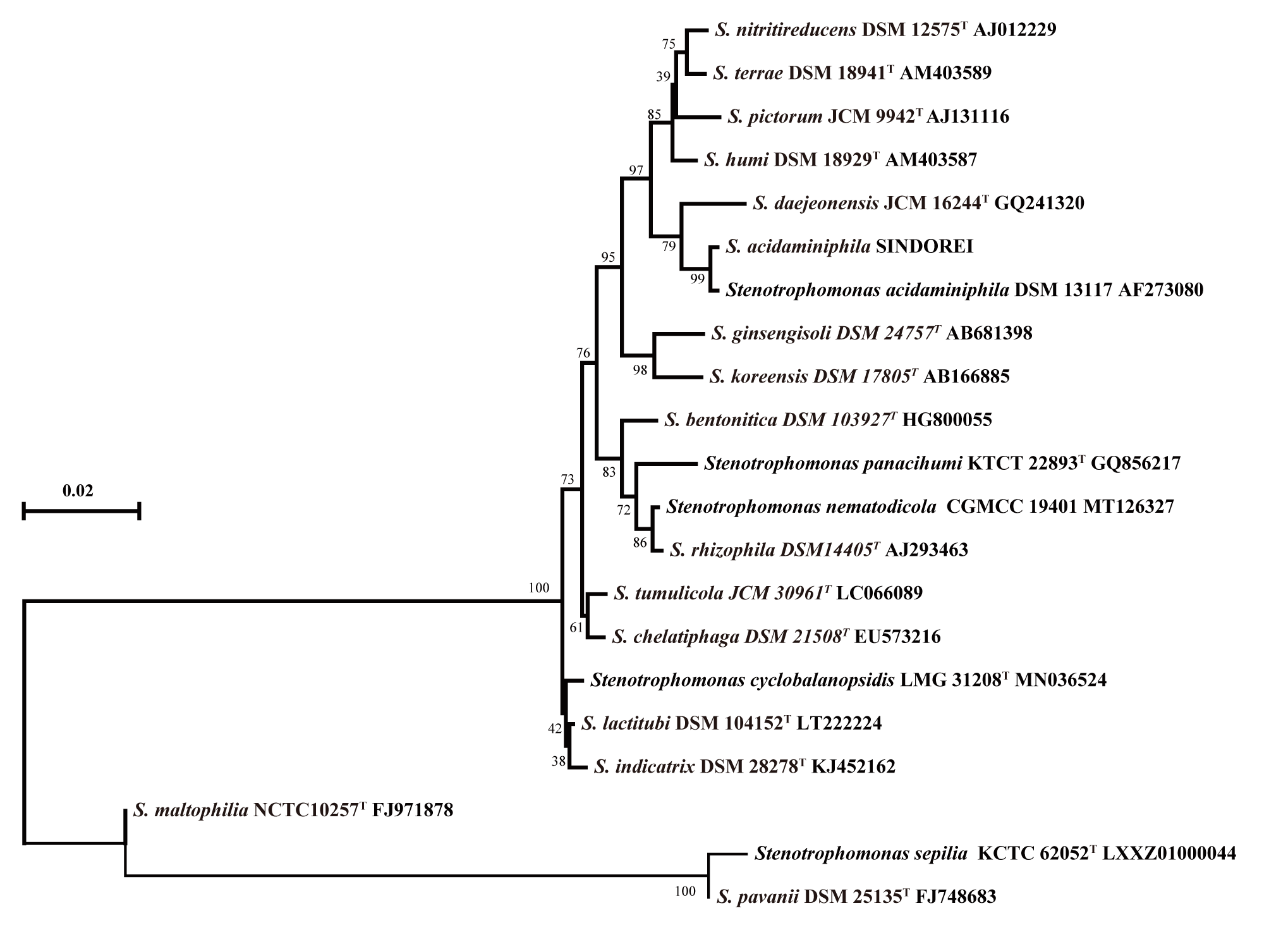


**Fig.S1 The phylogenetic tree based on the 16S rRNA gene sequences in genus Stenotrophomonas.** The numbers present on the branches of the tree represent the bootstrap values (based on 1000 replicates). The scale bar indicates 0.1 substitutions per nucleotide position.


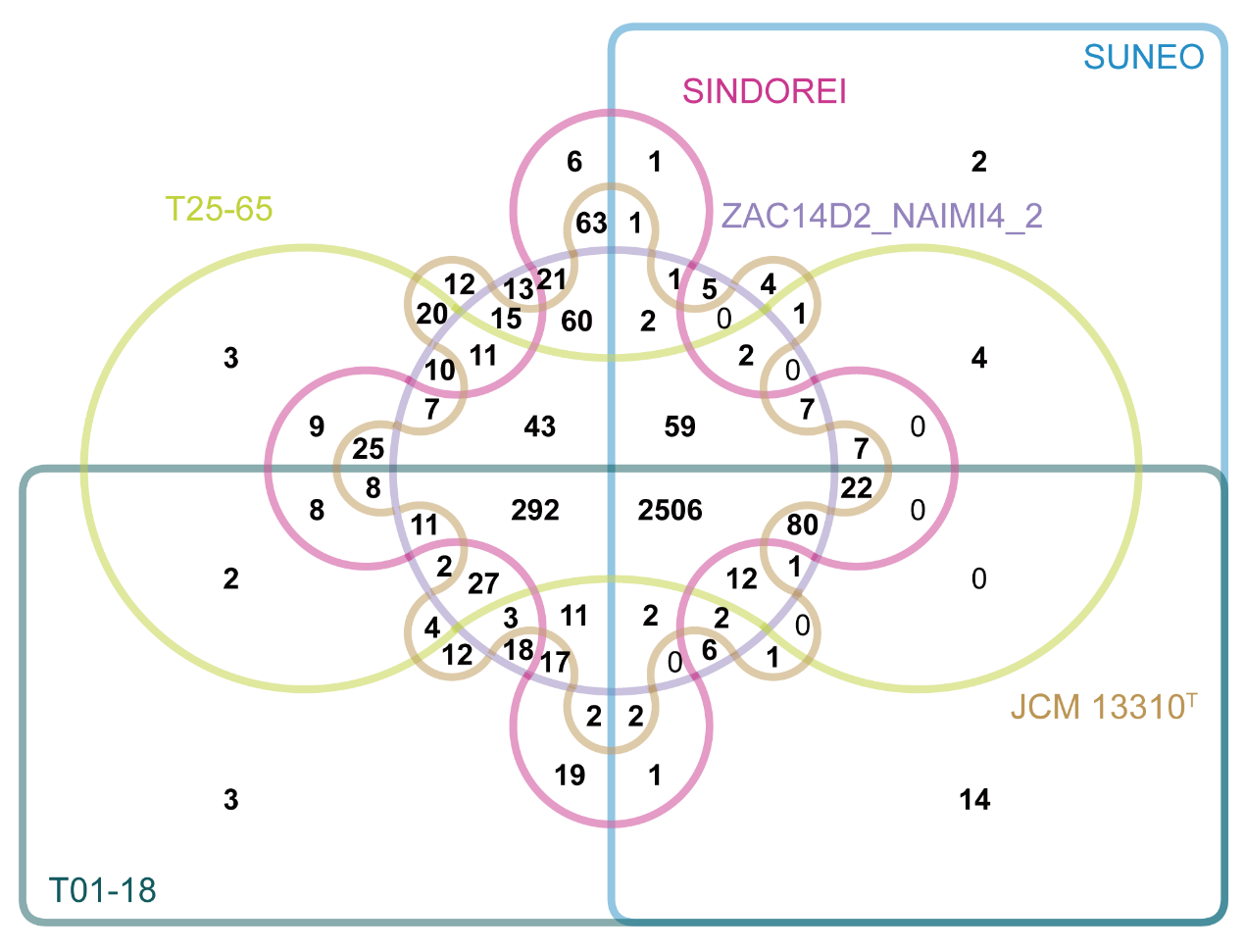


**Fig.S2 The venn diogram of orthogroups in *S. acidaminiphila*.**
